# Association Between Weekend Catch-Up Sleep and Obesity Among Working Adults: A Cross-Sectional Nationwide Population-Based Study

**DOI:** 10.3390/life15101562

**Published:** 2025-10-06

**Authors:** Wonseok Jeong, Min Ji Song, Ji Hye Shin, Ji Hyun Kim

**Affiliations:** 1 Department of Public Health, Graduate School, Seoul National University, Seoul 08826, Republic of Korea; wsjeong22@snu.ac.kr; 2 Department of Neurology, Korea University Guro Hospital, Korea University College of Medicine, Seoul 08308, Republic of Korea; songminji1034@gmail.com (M.J.S.); jhshin@kumc.or.kr (J.H.S.)

**Keywords:** sleep pattern, weekend catch-up sleep, general obesity, abdominal obesity, workers

## Abstract

Objectives: This study aimed to examine the association between weekend catch-up sleep (CUS) and obesity among Korean workers. Methods: Data were derived from the 2016–2023 Korean National Health and Nutrition Examination Survey (KNHANES), a nationally representative dataset. The final analytic sample comprised 17,208 Korean workers aged 26 to 64 years. General and abdominal obesity were defined as body mass index (BMI) ≥ 25 kg/m^2^ and waist circumference ≥ 90 cm for men and ≥85 cm for women, respectively. Sleep patterns were categorized into sufficient sleep, weekend CUS, and insufficient sleep. Multivariable logistic regression analyses were performed to evaluate associations between sleep patterns and obesity, adjusting for demographic, socioeconomic, and health-related variables. Results: Compared to individuals with sufficient sleep, those with weekend CUS showed increased odds of general obesity (adjusted odds ratio [AOR] = 1.21) and abdominal obesity (AOR = 1.18). The insufficient sleep group had even higher odds for both general obesity (AOR = 1.23) and abdominal obesity (AOR = 1.33). Conclusions: Insufficient sleep is significantly associated with increased risks of both general and abdominal obesity among Korean workers. While weekend CUS may offer partial mitigation of obesity risk, it should not be considered a substitute for regular, adequate sleep. Longitudinal studies are warranted to further explore causal relationships between sleep patterns and obesity in working populations.

## 1. Introduction

Obesity, characterized by excessive fat accumulation, is a well-established risk factor for increased morbidity, including cardiovascular disease, musculoskeletal disorders, and several types of cancer [1,2]. Despite growing awareness of these consequences, the global prevalence of obesity continues to rise [3]. In the United States, for example, the prevalence of adult obesity increased from 30.5% in 1999–2000 to 41.9% in 2017–2020 [4]. Similar upward trends have been observed in Asian countries; in South Korea, the adult obesity rate rose from 30.2% in 2012 to 38.4% in 2021, with increases noted across all age groups [5]. According to the World Obesity Atlas 2023, approximately 38% of the global population aged over five years is classified as overweight or obese, prompting the World Health Organization (WHO) to recognize obesity as a global epidemic [3,5].

Sleep plays a fundamental role in maintaining overall health and physiological homeostasis [6]. Insufficient sleep has been linked to a broad spectrum of adverse health outcomes, including diabetes mellitus, hypertension, cardiovascular diseases, stroke, coronary heart disease, and obesity [7]. Previous studies have demonstrated that each additional hour of sleep is associated with a 0.40-unit reduction in body mass index (BMI) among men [8], and a meta-analysis confirmed that short sleep duration significantly increases obesity risk [9]. In particular, short sleep duration has been strongly associated with abdominal obesity [10], which is considered a more robust indicator of metabolic risk than BMI alone [11,12]. These findings underscore the importance of adequate sleep as an integral component of a healthy lifestyle, especially amidst the growing obesity epidemic.

Although sleep quality and duration are influenced by various factors, occupational and social demands are among the most common contributors to sleep restriction. Long working hours and chronic job-related stress are major drivers of insufficient sleep and poor sleep quality [13,14]. Consequently, reduced sleep duration is more pronounced among working-age men [15], especially those employed in shift-based or irregular work schedules [16]. These patterns are particularly prominent in East Asian countries such as South Korea and Japan, where cultural norms prioritize extended work hours. South Koreans report some of the longest average working hours among developed countries [17], while their mean sleep duration is the shortest, at 461 min compared with the OECD average of 505 min during the same period [18]. In Japan, long working hours have been associated with serious health consequences, including death from overwork (karoshi) and mental or physical illnesses [19].

Due to long working hours and high levels of work-related stress during the weekdays, many individuals compensate for insufficient sleep by extending their sleep on weekends, which is a behavior commonly referred to as weekend catch-up sleep (CUS) [20]. In South Korea, this behavior is particularly evident on Saturdays and has become increasingly prevalent among adults [21]. Although the association between overall sleep duration and obesity is well established, research specifically exploring the relationship between weekend CUS and obesity remains scarce, particularly among working populations. While some studies have reported potential health benefits of CUS [20,22], its specific influence on obesity risk has not been comprehensively examined.

This study investigated the association between sleep patterns—including weekend CUS—and both general and abdominal obesity among Korean workers, using BMI and waist circumference as outcome measures. We hypothesized that weekend CUS would confer health benefits, manifesting as lower BMI and waist circumference among workers with insufficient weekday sleep.

## 2. Materials and Methods

### 2.1. Study Design

This study utilized data from the 2016–2023 cycles of the Korean National Health and Nutrition Examination Survey (KNHANES), a nationally representative survey conducted by the Korea Disease Control and Prevention Agency (KDCA) [23]. KNHANES gathers comprehensive information on health status and behaviors, including smoking, dietary habits, and physical activity levels, through a combination of self-administered questionnaires, face-to-face interviews, and standardized physical examinations conducted by trained personnel. The survey protocol was exempted from ethics review by the Institutional Review Board of the KDCA (IRB No. 2018-01-03-C-A), in accordance with the Bioethics and Safety Act of 2015, as the data are publicly available (https://knhanes.kdca.go.kr/knhanes/main.do, accessed on 7 March 2025).

The survey employs a complex, stratified, multistage probability cluster sampling design to ensure national representativeness of the non-institutionalized civilian population in South Korea. Sampling proceeds in two stages: primary sampling units (PSUs) are generated based on stratification by sex, 26 age categories, and 24 housing/residential classifications. Subsequently, 20 households are randomly selected from each PSU. Written informed consent is obtained from all participants. Owing to its rigorous methodology, KNHANES is regarded as a robust and generalizable data source for population-level research in South Korea.

**Figure 1:**
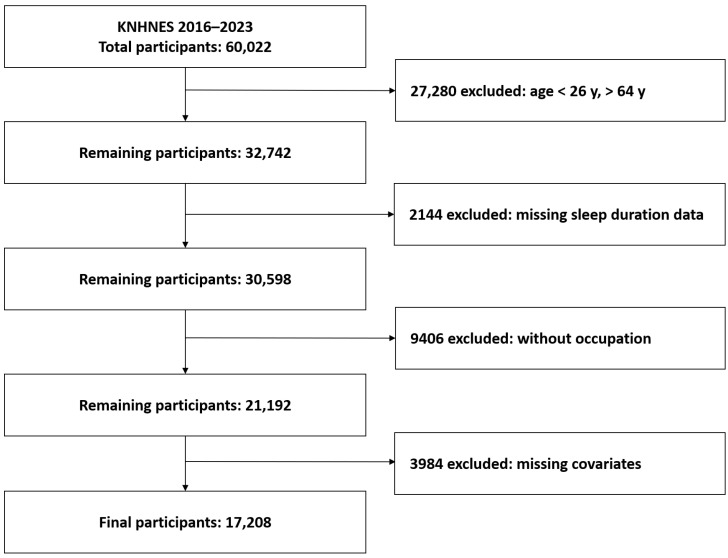
Flowchart depicting enrollment process of the participants.

Of the 60,022 participants surveyed from 2016 to 2023, we excluded 27,280 participants aged under 26 or over 65 to focus on the working-age population. We further excluded 2144 participants with missing data on sleep duration, 9406 without occupational data, and 3984 with missing information on key covariates including regular exercise, breakfast and eating-out frequency, diabetes, and hypertension. The final analytic sample comprised 17,208 participants (Figure 1).

### 2.2. Main Variables

The primary variable was sleep pattern, categorized as sufficient sleep, weekend CUS, and insufficient sleep. Classification was based on self-reported weekday and weekend bedtimes and wake times. Average weekday and weekend sleep durations were calculated. Participants who reported ≥7 h of sleep on weekdays were categorized as having sufficient sleep, in line with National Sleep Foundation guidelines for adults aged 26–64 [24]. Among participants with <7 h of weekday sleep, those reporting longer sleep duration on weekends were classified as CUS, while those without additional weekend sleep were defined as having insufficient sleep.

The outcomes were general obesity and abdominal obesity. General obesity was defined as BMI ≥ 25 kg/m^2^, according to the WHO Asian-specific criteria [25]. Abdominal obesity was defined as waist circumference (WC) of ≥90 cm for men and ≥85 cm for women, based on the criteria established by the Korean Society for the Study of Obesity (KSSO) [26].

### 2.3. Secondary Variables

Demographic variables included age (categorized as 26–39, 40–49, and 50–64 years), and sex. Socioeconomic variables encompassed education level (middle school or less, high school, college or higher), place of residence (urban or rural), and occupation. Occupations were reclassified, according to the Korean Standard Classification of Occupations, into white-collar (office/managerial), pink-collar (service/sales), and blue-collar (manual labor, agriculture, forestry, fisheries, and military) [27].

Health-related variables included drinking level (heavy: ≥2 days/week, moderate: ≤1 day/week, light: approximately once/month), regular exercise (defined per WHO guidelines as ≥75 min/week of vigorous or ≥150 min/week of moderate activity) [28], frequency of breakfast and eating out, and physician-diagnosed diabetes or hypertension.

### 2.4. Statistical Analysis

Descriptive statistics summarized participants’ characteristics. Group differences in categorical variables were tested using chi-square analysis; when the overall test was significant, post hoc pairwise comparisons were conducted with Bonferroni correction. For continuous variables, group differences were assessed with ANOVA after confirming normality (Kolmogorov–Smirnov test) and homogeneity of variances (Levene test). When ANOVA was significant, Tukey’s post hoc test was further applied. Associations between sleep patterns and obesity outcomes were assessed using multivariable logistic regression to estimate adjusted odds ratios (AORs) and 95% confidence intervals (CIs). All models were adjusted for demographic, socioeconomic, and health-related covariates. Statistical significance was defined as a two-tailed *p*-value < 0.05. Analyses were conducted using SAS software (version 9.4; SAS Institute Inc., Cary, NC, USA).

## 3. Results

Table 1 presents the general characteristics of the study population. Among the 17,208 participants, 37.1% (*n* = 6390) were classified as obese based on BMI, while 31.5% (*n* = 5415) met the criteria for abdominal obesity based on waist circumference. Regarding sleep patterns, 56.9% of participants reported sufficient sleep, 25.9% reported weekend CUS, and 17.2% reported insufficient sleep. Notably, the prevalence of both general and abdominal obesity was highest among individuals with insufficient sleep (41.6% and 37.9%, respectively). Post hoc pairwise analyses showed significant differences across sleep pattern groups for all variables except breakfast frequency, place of residence, and sex. For breakfast frequency, no difference was observed between participants consuming breakfast 1–4 times per week and those consuming it less than once per week. For place of residence, no difference was detected between the insufficient and sufficient sleep groups. Similarly, for sex, no difference was observed between the insufficient and CUS groups.

Table 2 displays the associations between sleep patterns and general obesity (BMI ≥ 25 kg/m^2^) after adjusting for demographic, socioeconomic, and health-related covariates. Compared with the sufficient sleep group, the adjusted odds of general obesity were higher among participants with weekend CUS (AOR = 1.21, 95% CI = 1.12–1.31) and highest among those with insufficient sleep (AOR = 1.23, 95% CI = 1.13–1.34). When comparing the insufficient sleep group directly to the weekend CUS group, excluding the sufficient sleep group, the difference in obesity risk was not statistically significant (AOR = 1.05, 95% CI: 0.95–1.17), although the trend remained in the anticipated direction.

In terms of demographic and lifestyle variables, participants aged 40–49 years, those employed in white-collar or blue-collar occupations, individuals with higher educational attainment, married participants, and individuals in lower-income groups exhibited significantly greater odds of obesity compared to their counterparts. Unexpectedly, higher breakfast frequency was positively associated with general obesity (≥5 times/week: AOR = 1.21, 95% CI = 1.10–1.32; 1–4 times/week: AOR = 1.19, 95% CI = 1.10–1.29). Regular exercise was not significantly associated with general obesity (AOR = 1.02, 95% CI = 0.96–1.09).

Associations between sleep patterns and abdominal obesity, defined by waist circumference, were also presented in Table 2. The direction and strength of associations were consistent with those for general obesity. Compared with the sufficient sleep group, the odds of abdominal obesity were significantly higher in both the CUS (AOR = 1.18, 95% CI = 1.09–1.27) and insufficient sleep groups (AOR = 1.33, 95% CI = 1.21–1.45). A direct comparison between the insufficient group and CUS group, excluding the sufficient sleep group, indicated a significantly higher risk of abdominal obesity in the insufficient sleep group (AOR = 1.15, 95% CI = 1.04–1.28). The associations between covariates and abdominal obesity generally mirrored those observed for general obesity. However, unlike general obesity, regular exercise was inversely associated with abdominal obesity (AOR = 0.85, 95% CI = 0.79–0.91), suggesting a differential impact of regular exercise on fat distribution compared to overall weight status.

Table 3 presents subgroup analyses of the association between sleep patterns and general and abdominal obesity by occupation. Among white-collar workers, the adjusted odds of general obesity were higher in CUS group (AOR = 1.30, 95% CI = 1.16–1.45) and highest in insufficient sleep group (AOR = 1.36, 95% CI = 1.18–1.58). A similar pattern was observed for abdominal obesity, with a stronger effect of insufficient sleep (AOR = 1.47, 95% CI = 1.27–1.71) than CUS (AOR = 1.22, 95% CI = 1.09–1.37). Among blue-collar workers, sleep patterns were not significantly associated with general obesity; however, both insufficient sleep (AOR = 1.26, 95% CI = 1.09–1.45) and CUS (AOR = 1.17, 95% CI = 1.02–1.35) were risk factors for abdominal obesity. For pink-collar workers, no consistent differences were observed across sleep patterns, except for a higher risk of general obesity in the insufficient sleep versus sufficient sleep (AOR = 1.23, 95% CI = 1.02–1.47).

## 4. Discussion

Obesity remains a growing public health challenge globally, yet its modifiable risk factors, particularly those related to sleep behavior in working populations, remain insufficiently explored. This study investigated the relationship between weekend CUS and obesity among Korean workers, using a large, nationally representative dataset. Our findings indicate that insufficient weekday sleep is significantly associated with increased risk of both general and abdominal obesity, whereas individuals who engaged in weekend CUS exhibited a modestly lower risk of obesity compared to those who did not compensate for sleep loss. These results suggest a potential, though partial, mitigating effect of compensatory sleep on obesity.

Recent research has suggested that weekend CUS may confer health benefits, including reduced risk of metabolic syndrome [29], type 2 diabetes [30], and depression [20]. While the association between short sleep duration and increased obesity risk has been consistently reported [31,32], evidence regarding the protective effects of CUS remains limited and inconsistent, particularly in adult working populations. Many existing studies on the link between weekend CUS and obesity have focused on children and adolescents [33,34,35], whereas only a few studies examined this relationship in adults, yielding inconsistent findings. Some studies reported an inverse association between weekend CUS and BMI [36,37], while another reported a positive association [38]. These discrepancies may be due to differences in measurement methods for obesity and study populations. Importantly, many previous studies relied solely on BMI to define obesity, which does not differentiate between fat and lean mass or capture regional fat distribution [39]. This can lead to misclassification, particularly among physically active individuals or those with high muscle mass. Our study addressed this limitation by including waist circumference, a more specific indicator of visceral adiposity and cardiometabolic risk. Notably, the association between insufficient sleep and obesity was more pronounced for abdominal obesity rather than general obesity, highlighting the importance of incorporating both BMI and waist circumference in obesity-related research. Moreover, all of the included studies involved non-working populations, for whom the concept of weekend CUS may be less applicable. In individuals without structured work schedules, weekend sleep extension may reflect irregular sleep patterns rather than compensation for weekday sleep deprivation.

Our findings underscore the critical importance of structured daily schedules when evaluating the impact of weekend CUS on obesity. Similar to working adults, adolescents are subject to rigid daily routines and substantial academic demands, often resulting in insufficient sleep during weekdays. For instance, early school start times and heavy homework burdens are well-established contributors to chronic weekday sleep deprivation in this group [40]. These parallels between students and working adults suggest that the effects of CUS may be more pronounced in populations with regimented schedules. Furthermore, the differing associations between regular exercise and the two types of obesity observed in our study highlight the limitations of using BMI as the sole metric of obesity. Specifically, the finding that individuals who reported engaging in regular exercise exhibited higher odds of obesity when classified by BMI underscores the inadequacy of BMI in accurately reflecting body fat composition [41]. In contrast, when abdominal obesity—measured by waist circumference—was used as the outcome, the results were more consistent with established evidence [42], emphasizing the necessity of incorporating additional anthropometric indicators to better assess obesity-related health risks. Our study investigated the relationship between CUS and obesity, as defined by both BMI and waist circumference, among employed adults. Consistent with previous studies, sleep deprivation was associated with increased risk of both general and abdominal obesity. Notably, the adverse associations linked to insufficient sleep were attenuated among individuals who engaged in weekend CUS, across both obesity measures. The association between sleep patterns and obesity was particularly pronounced in the case of abdominal obesity, underscoring the importance of evaluating fat distribution rather than relying solely on general obesity indices in sleep-obesity research.

Subgroup analyses indicated that associations between sleep patterns and obesity differed by occupation. Among white-collar workers, insufficient sleep was strongly associated with higher odds of both general and abdominal obesity, whereas weekend CUS showed a modest attenuation of risk. This pattern may reflect the sedentary nature of white-collar work [43], in which limited physical activity could exacerbate the metabolic consequences of short sleep [44]. In contrast, among blue-collar workers, sleep patterns were not associated with general obesity; however, both insufficient sleep and CUS were associated with abdominal obesity. Given the higher physical demands of blue-collar jobs, general obesity risk may be less sensitive to sleep restriction, whereas abdominal adiposity—more closely linked to stress and circadian disruption—may remain affected [45,46]. For pink-collar workers, sleep patterns were not consistently related to obesity risk, except for higher odds of general obesity with insufficient versus sufficient sleep. These findings suggest that occupational characteristics (e.g., activity level, schedule regularity, and customer-facing tasks) may modify the impact of sleep on metabolic outcomes.

Several biological and physiological mechanisms may explain the observed association between sleep duration and obesity. Adequate sleep is essential for maintaining homeostasis across multiple systems that regulate energy balance, metabolism, and body weight. One prominent mechanism involves the modulation of appetite-related hormones. Sleep restriction has been shown to increase circulating levels of ghrelin, a hormone that stimulates appetite, while concurrently decreasing levels of leptin, a hormone involved in promoting satiety [47]. This hormonal imbalance enhances subjective hunger and often leads to increased caloric intake and a preference for energy-dense, high-fat foods, thereby promoting weight gain.

Sleep deprivation also affects neural pathways involved in reward processing and impulse control. Neuroimaging studies have shown that acute sleep deprivation increases amygdala activation in response to energy-dense food cues, indicating stronger cravings driven by hedonic, rather than homeostatic, mechanisms [48,49]. Another critical mechanism involves the impact of sleep loss on glucose metabolism and insulin sensitivity. Even in healthy adults, insufficient sleep impairs glucose regulation and reduces insulin sensitivity in peripheral tissues [50]. Chronic sleep deprivation may thereby contribute to the development of type 2 diabetes and long-term metabolic dysregulation. These alterations can disrupt overall energy homeostasis and enhance fat accumulation, even in the absence of significant changes in dietary intake or physical activity levels [50].

This study has several important limitations. First, all variables—including sleep duration—were self-reported, which introduces potential recall and misclassification bias. Second, the cross-sectional design precludes causal inference and limits assessment of long-term effects of sleep patterns on obesity. Third, due to data constraints, granular work-related measures beyond broad occupational categories were unavailable for analysis. Future studies should incorporate objective sleep measures (e.g., actigraphy), adopt longitudinal designs to clarify causal pathways, and collect more detailed occupational and lifestyle data to better capture contextual influences on sleep and obesity. Despite these limitations, the study offers notable strengths. First, it utilized data from the KNHANES, a nationally representative dataset collected through stratified, multistage cluster sampling. This enhances both the statistical robustness and the generalizability of the findings. The inclusion of eight years of KNHANES data allowed for a large, comprehensive sample, addressing limitations of prior studies that commonly relied on fewer years of data. Second, this study is the first to investigate the potential health benefits of weekend CUS in relation to both general and abdominal obesity. Third, it is among the few studies to explore the effects of compensatory sleep on health outcomes specifically within a working adult population.

## 5. Conclusions

This study investigated the associations between sleep patterns including weekend CUS and both general and abdominal obesity among Korean workers using a nationally representative survey. Insufficient weekday sleep was associated with higher risks for general and abdominal obesity, whereas extending sleep on weekends (CUS) was linked to modest reductions in both outcomes. These findings partly support our hypothesis that CUS mitigates the adverse effects of weekday sleep deprivation. However, CUS should not be viewed as a substitute for regular sufficient sleep, as it reflects a short-term compensatory behavior rather than a sustainable protective strategy. Future longitudinal studies are needed to clarify the causal relationships between varying sleep patterns and obesity, particularly in working populations.

## Figures and Tables

**Table 1:** General characteristics of the study population.

		Sleep Patterns				
Variables	Total (*n* = 17,208)	Insufficient Sleep (*n* = 2954)	Catch-Up Sleep (*n* = 4464)	Sufficient Sleep (*n* = 9790)	*p* Value	Effect Size
Sleep duration (h)	7.22 (1.21)	5.66 (0.70)	6.81 (0.99)	7.88 (0.86)	<0.001	0.481
General obesity					<0.001	0.057
Yes	6390 (37.1)	1228 (41.6)	1752 (39.2)	3410 (34.8)		
No	10,818 (62.9)	1726 (58.4)	2712 (60.8)	6380 (65.2)		
Abdominal obesity					<0.001	0.068
Yes	5415 (31.5)	1120 (37.9)	1433 (32.1)	2862 (29.2)		
No	11,793 (68.5)	1834 (62.1)	3031 (67.9)	6928 (70.8)		
Age (y)					<0.001	0.130
26–39	4960 (28.8)	446 (15.1)	1509 (33.8)	3005 (30.7)		
40–49	5010 (29.1)	738 (25.0)	1446 (32.4)	2826 (28.9)		
50–64	7238 (42.1)	1770 (59.9)	1509 (33.8)	3959 (40.4)		
Sex					<0.001	0.042
Male	8909 (51.8)	1630 (55.2)	2383 (53.4)	4896 (50.0)		
Female	8299 (48.2)	1324 (44.8)	2081 (46.6)	4894 (50.0)		
Occupation classification					<0.001	0.072
White-collar	8169 (47.5)	1107 (37.5)	2374 (53.2)	4688 (47.9)		
Blue-collar	5514 (32.0)	1117 (37.8)	1303 (29.2)	3094 (31.6)		
Pink-collar	3525 (20.5)	730 (24.7)	787 (17.6)	2008 (20.5)		
Education level					<0.001	0.091
Middle school or less	1969 (11.4)	514 (17.4)	309 (6.9)	1146 (11.7)		
High school	5253 (30.5)	1034 (35.0)	1270 (28.4)	2949 (30.1)		
College or over	9986 (58.0)	1406 (47.6)	2885 (64.6)	5695 (58.2)		
Marital status					<0.001	0.079
Married	12,935 (75.2)	2293 (77.6)	3166 (70.9)	7476 (76.4)		
Separated or divorced	1495 (8.7)	369 (12.5)	361 (8.1)	765 (7.8)		
Unmarried	2778 (16.1)	292 (9.9)	937 (21.0)	1549 (15.8)		
Place of residence					<0.001	0.049
Rural	9749 (56.7)	1744 (59.0)	2348 (52.6)	5657 (57.8)		
Urban	7459 (43.3)	1210 (41.0)	2116 (47.4)	4133 (42.2)		
Household income level					<0.001	0.048
Low	911 (5.3)	220 (7.4)	167 (3.7)	524 (5.4)		
Lower middle	3630 (21.1)	689 (23.3)	859 (19.2)	2082 (21.3)		
Upper middle	5640 (32.8)	939 (31.8)	1490 (33.4)	3211 (32.8)		
High	7027 (40.8)	1106 (37.4)	1948 (43.6)	3973 (40.6)		
Breakfast frequency (/week)					<0.001	0.058
≥5	3434 (20.0)	501 (17.0)	1047 (23.5)	1886 (19.3)		
1–4	4529 (26.3)	708 (24.0)	1304 (29.2)	2517 (25.7)		
<1	9245 (53.7)	1745 (59.1)	2113 (47.3)	5387 (55.0)		
Eating out frequency (/week)					<0.001	0.082
5–7	9961 (57.9)	1491 (50.5)	2951 (66.1)	5519 (56.4)		
1–4	4941 (28.7)	919 (31.1)	1081 (24.2)	2941 (30.0)		
<1	2306 (13.4)	544 (18.4)	432 (9.7)	1330 (13.6)		
Drinking level					<0.001	0.040
Heavy	1333 (7.7)	317 (10.7)	278 (6.2)	738 (7.5)		
Moderate	7977 (46.4)	1314 (44.5)	2108 (47.2)	4555 (46.5)		
Light	7898 (45.9)	1323 (44.8)	2078 (46.6)	4497 (45.9)		
Regular exercise					<0.001	0.036
Yes	7970 (46.3)	1393 (47.2)	2186 (49.0)	4391 (44.9)		
No	9238 (53.7)	1561 (52.8)	2278 (51.0)	5399 (55.1)		
Diabetes					<0.001	0.054
Yes	1059 (6.2)	266 (9.0)	244 (5.5)	549 (5.6)		
No	16,149 (93.8)	2688 (91.0)	4220 (94.5)	9241 (94.4)		
Hypertension					<0.001	0.028
Yes	1029 (6.0)	220 (7.4)	259 (5.8)	550 (5.6)		
No	16,179 (94.0)	2734 (92.6)	4205 (94.2)	9240 (94.4)		

Values are presented as number (%) or mean (standard deviation), as appropriate. Effect sizes are reported as Cramer’s V for chi-square tests and ηp^2^ for ANOVA. Occupational categories were defined as white-collar (office/managerial), pink-collar (service/sales), and blue-collar (manual labor, agriculture, forestry, fisheries, and military). Drinking level was defined as heavy (≥2 days/week), moderate (≤1 day/week), and light (approximately once/month). Regular exercise is defined according to WHO guidelines as ≥75 min/week of vigorous activity or ≥150 min/week of moderate activity.

**Table 2:** Associations between sleep patterns and general and abdominal obesity.

Variables	General Obesity		Abdominal Obesity	
	AOR (95% CI)	*p* Value	AOR (95% CI)	*p* Value
Sleep pattern				
Insufficient sleep	1.23 (1.13–1.35) ***	<0.001	1.33 (1.21–1.45) ***	<0.001
Catch-up sleep	1.21 (1.12–1.31) ***	<0.001	1.18 (1.09–1.27) ***	<0.001
Sufficient sleep	1.00		1.00	
Age (years)				
26–39	1.11 (1.00–1.23)	0.052	0.92 (0.83–1.02)	0.124
40–49	1.11 (1.02–1.22) *	0.015	0.94 (0.86–1.03)	0.164
50–64	1.00		1.00	
Sex				
Male	2.47 (2.29–2.66) ***	<0.001	2.27 (2.10–2.45) ***	<0.001
Female	1.00		1.00	
Occupation classification				
White-collar	0.85 (0.78–0.93) ***	<0.001	0.89 (0.81–0.98) *	0.018
Blue-collar	0.82 (0.75–0.90) ***	<0.001	0.82 (0.74–0.90) ***	<0.001
Pink-collar	1.00		1.00	
Education level				
Middle school or less	1.44 (1.26–1.64) ***	<0.001	1.38 (1.21–1.58) ***	<0.001
High school	1.17 (1.07–1.27) ***	<0.001	1.16 (1.06–1.26) **	0.001
College or over	1.00		1.00	
Marital status				
Married	1.14 (1.03–1.26) **	0.009	1.19 (1.07–1.32) **	0.001
Separated or divorced	1.02 (0.87–1.19)	0.830	1.12 (0.95–1.31)	0.173
Unmarried	1.00		1.00	
Place of residence				
Rural	1.13 (1.06–1.21) ***	<0.001	1.10 (1.03–1.18) **	0.007
Urban	1.00		1.00	
Household income level				
Low	1.21 (1.04–1.42) *	0.015	1.23 (1.05–1.45) ***	<0.001
Lower middle	1.15 (1.05–1.25) **	0.003	1.18 (1.07–1.30) ***	<0.001
Upper middle	1.14 (1.06–1.23) ***	<0.001	1.14 (1.06–1.24) **	0.001
High	1.00		1.00	
Breakfast frequency (/week)				
≥5	1.21 (1.10–1.32) ***	<0.001	1.18 (1.08–1.30) ***	<0.001
1–4	1.19 (1.10–1.29) ***	<0.001	1.13 (1.04–1.23) **	0.003
<1	1.00		1.00	
Eating out frequency (/week)				
5–7	1.09 (0.98–1.21)	0.121	1.03 (0.93–1.16)	0.552
1–4	1.14 (1.02–1.27) *	0.022	1.12 (1.00–1.26)	0.052
<1	1.00		1.00	
Drinking level				
Heavy	0.93 (0.82–1.05)	0.243	0.90 (0.75–1.07)	0.220
Moderate	1.03 (0.96–1.10)	0.404	1.07 (0.98–1.18)	0.139
Light	1.00		1.00	
Regular exercise				
Yes	1.02 (0.96–1.09)	0.569	0.85 (0.79–0.91) ***	<0.001
No	1.00		1.00	
Diabetes				
Yes	2.38 (2.08–2.72) ***	<0.001	2.69 (2.36–3.08) ***	<0.001
No	1.00		1.00	
Hypertension				
Yes	1.89 (1.65–2.16) ***	<0.001	2.26 (1.97–2.58) ***	<0.001
No	1.00		1.00	

* *p* < 0.05; ** *p* < 0.01; *** *p* < 0.001. Occupational categories were defined as white-collar (office/managerial), pink-collar (service/sales), and blue-collar (manual labor, agriculture, forestry, fisheries, and military). Drinking level was defined as heavy (≥2 days/week), moderate (≤1 day/week), and light (approximately once/month). Regular exercise is defined according to WHO guidelines as ≥75 min/week of vigorous activity or ≥150 min/week of moderate activity. Abbreviations: CI, confidence interval; AOR, adjusted odds ratio.

**Table 3:** Associations between sleep patterns and general and abdominal obesity by occupation.

Variables	Insufficient Sleep		Catch-Up Sleep		Sufficient Sleep
	AOR (95% CI)		AOR (95% CI)		AOR
General obesity					
White-collar	1.36 (1.18–1.58) ***		1.30 (1.16–1.45) ***		1.00
Blue-collar	1.10 (0.95–1.26)		1.12 (0.98–1.28)		1.00
Pink-collar	1.23 (1.02–1.47) *		1.11 (0.93–1.33)		1.00
Abdominal obesity					
White-collar	1.47 (1.27–1.71) ***		1.22 (1.09–1.37) ***		1.00
Blue-collar	1.26 (1.09–1.45) **		1.17 * (1.02–1.35) *		1.00
Pink-collar	1.19 (0.99–1.44)		1.04 (0.86–1.25)		1.00

* *p* < 0.05; ** *p* < 0.01; *** *p* < 0.001. Abbreviations: CI, confidence interval; AOR, adjusted odds ratio.

## Data Availability

The dataset is publicly accessible (https://knhanes.kdca.go.kr/knhanes/main.do, accessed on 7 March 2025).
